# Sexual dysfunction in women with hypermobile Ehlers–Danlos syndrome and hypermobility spectrum disorders: an online community-based study

**DOI:** 10.1093/rap/rkaf023

**Published:** 2025-02-27

**Authors:** Emily Fuster, Omid Mirmosayyeb, Svetlana Blitshteyn

**Affiliations:** Department of Neurology, University of Buffalo Jacobs School of Medicine and Biomedical Sciences, Buffalo, NY, USA; Department of Neurology, University of Buffalo Jacobs School of Medicine and Biomedical Sciences, Buffalo, NY, USA; Department of Neurology, University of Buffalo Jacobs School of Medicine and Biomedical Sciences, Buffalo, NY, USA; Dysautonomia Clinic, Williamsville, NY, USA

**Keywords:** hypermobility spectrum disorders (HSD), hypermobile Ehlers–Danlos syndrome, sexual dysfunction, autonomic dysfunction, women’s health, case–control study

## Abstract

**Objectives:**

Hypermobility spectrum disorders (HSD) and hypermobile Ehlers–Danlos syndrome (h-EDS) are connective tissue disorders associated with joint hypermobility, pain, fatigue and autonomic dysfunction. We sought to assess sexual function in women with h-EDS/HSD.

**Methods:**

In this cross-sectional community-based case–control study, women with h-EDS/HSD completed the following online questionnaires: Female Sexual Function Index (FSFI), 31-item Composite Autonomic Symptom Score (COMPASS-31), Beck Depression Inventory-II (BDI-II) and an additional short form with questions pertaining to comorbidities and sexual activity. Scores were compared with those of healthy female controls.

**Results:**

A total of 84 women with h-EDS/HSD [mean age 37.1 years (s.d. 8.4)] and 75 healthy women [mean age 29.79 years (s.d. 5.38)] completed the questionnaires. Of these, 75% were diagnosed with h-EDS, 25% with HSD and 58% had concurrent postural orthostatic tachycardia syndrome. A majority of women with h-EDS/HSD (52%) did not engage in any sexual activity, and only 25% reported having sexual intercourse with a partner in the past 6 months. The mean COMPASS-31 score was 51.5 (s.d. 13.8), mean BDI-II score was 24.6 (s.d. 11.4) and mean FSFI score was 15.3 (s.d. 7.9) in the patient group. Compared with healthy controls, women with h-EDS/HSD had decreased FSFI scores in the subdomains of desire, arousal, lubrication, orgasm and sexual satisfaction. Neither BDI-II nor COMPASS-31 scores were predictive of the FSFI score.

**Conclusion:**

Compared with healthy women, we found significant sexual dysfunction in women with h-EDS/HSD, which did not correlate with depressive or autonomic symptoms in this cohort. Given its health implications, sexual dysfunction represents a significant unmet need that calls for development of targeted diagnostic and therapeutic approaches in the care of women with h-EDS/HSD.

Key messages

- We found significant sexual dysfunction in women with h-EDS/HSD, which did not correlate with autonomic or depressive symptoms.
- 52% of women did not engage in any sexual activity over the past 6 months, with fatigue, pain and a lack of libido reported as common reasons for sexual inactivity.
- Compared with women with POTS from our prior study, women with h-EDS/HSD reported greater sexual dysfunction overall and across all domains despite having comparable autonomic symptom burden.

## Introduction

Hypermobility spectrum disorders (HSD) are connective tissue disorders that cause joint hypermobility, instability, injury and pain. Other problems such as fatigue, autonomic dysfunction, headaches, gastrointestinal problems and other systemic manifestations are often seen in HSD [1]. Currently the spectrum of manifestations and genetic predisposition in HSD is poorly understood [2]. In 2017 the Ehlers–Danlos Society published an update on the diagnostic approach to these disorders, with defined diagnostic criteria for hypermobile Ehlers–Danlos syndrome (h-EDS) and introduced HSD for patients not meeting the diagnostic criteria for h-EDS [3].

With a loosely predicted prevalence approaching 4% of the general population, underdiagnosis has historically been common [4]. Other sources posit that h-EDS and HSD are the most common symptomatic joint hypermobility conditions seen in clinical practice [5]. Continued underrecognition and misdiagnosis of these disorders is fuelled by the lack of clinician familiarity and experience with the disorders, a highly variable clinical presentation and the absence of confirmatory diagnostic testing guidelines. Lastly, the high rate of extra-articular manifestations and the multisystemic nature of symptoms, signs and comorbidities adds to the challenge of identifying and diagnosing patients with h-EDS/HSD [1].

Research on h-EDS/HSD has been limited, especially as it applies to women’s health. One study reported gynaecologic and obstetric complications in women with h-EDS/HSD, including a higher prevalence of vulvodynia than in the general population [6]. To our knowledge, there have been no studies conducted to date on the topic of sexual health in women with h-EDS/HSD. We sought to determine whether sexual dysfunction exists in women with h-EDS/HSD compared with healthy controls and whether autonomic or depressive symptoms might predict sexual dysfunction. We also sought to delineate the nature of sexual dysfunction and reasons for sexual inactivity in women with h-EDS/HSD.

## Methods

In this community-based case–control study, we recruited participants to complete several online validated questionnaires and a short form. This study was approved by the University of Buffalo Institutional Review Board (STUDY00006037).

Study participants were recruited via invitation of the investigator (S.B.) to patients and announcements made on several social media platforms, including patient support groups on Facebook and X (formerly Twitter). Inclusion criteria were a self-reported diagnosis of h-EDS or HSD made by a physician, age ≥19  years, not currently pregnant and able to read English. Healthy controls were similarly recruited via word of mouth and social media platforms with the following inclusion criteria: age ≥19  years; without major medical conditions such as hypertension, diabetes, rheumatoid arthritis (RA), postural orthostatic tachycardia syndrome (POTS), connective tissue disorders, chronic fatigue or chronic pain; not currently pregnant; and able to read English.

All questionnaires were completed online via a secure website after the informed consent was signed by each participant electronically.

The study participants with h-EDS and HSD completed the following questionnaires: 31-item Composite Autonomic Symptom Score (COMPASS-31), which is a validated questionnaire that assesses the severity of autonomic dysfunction [7]; Female Sexual Function Index (FSFI), a validated sexual dysfunction questionnaire [8]; Beck Depression Inventory-II (BDI-II), which has been used in previous studies assessing sexual dysfunction and chronic illness [9–11], with higher total scores indicating more severe depression; and one additional short form that consisted of yes/no questions asking about comorbid diagnoses and sexual activity as well as a short response question that followed up on sexual inactivity.

The controls completed the FSFI and BDI-II. There were no missing data or unanswered questions from the online questionnaires completed by either group.

### Statistical analysis

The statistical analyses were conducted using SPSS version 28 for Windows (IBM, Armonk, NY, USA). Data distribution was evaluated using the Kolmogorov–Smirnov test along with Q-Q and P-P plots. Categorical data were expressed as frequencies and percentages, while continuous data were presented as mean (s.d.) for normally distributed data or median [interquartile range (IQR)] for non-normally distributed data. Comparisons of baseline characteristics between HSD patients and healthy controls (HCs) were made using Student’s *t*-test for continuous normally distributed data and equivalent non-parametric tests were used otherwise. Correlations between the study variables were determined using Pearson’s correlation coefficients. Variables differing between HSD patients and HCs were subjected to univariate linear regression analyses to determine their associations with female sexual dysfunction. Subsequently these variables were included in the multivariate linear regression models, employing the enter method to adjust for potential confounders. Similarly, variables differing between HSD patients and HCs were analysed through univariate and multivariate logistic regression models to explore their associations with sexual dysfunction in HSD patients, using the enter method for controlling confounding variables. *P*-values <0.05 were considered statistically significant. A conservative target sample size of at least 50 participants per group was set. An a priori power calculation for a difference in FSFI scores was not undertaken, but for reference, this sample size provides 80% power at a 5% significance level to detect a standardized mean difference between groups of at least 0.57. This effect size is considered moderate to large, aligning with our expectations.

## Results

### Patient characteristics

A total of 84 female h-EDS/HSD patients, mean age 37.1 years (s.d. 8.4), and 75 healthy female controls, mean age 29.79 years (s.d. 5.38), participated (Cohen’s *d* = 1.027, *P* < 0.001). Of these, 25% were diagnosed with HSD and 75% were diagnosed with h-EDS by their physician. A total of 58% reported a concurrent diagnosis of POTS and 98.8% of patients reported having chronic pain. Details of the participants’ characteristics and mean scores are presented in Table 1.

**Table 1.** Overview of the results

Characteristics	Women with h-EDS/HSD (*n* = 84)	Healthy women (*n* = 75)	Test statistics (Cohen’s *d*)	*P*-value
Age, years, mean (s.d.)	37.15 (8.45)	29.79 (5.38)	1.027	<0.001
FSFI, mean (s.d.)	15.34 (7.93)	23.24 (7.21)	−1.038	<0.001
Desire	2.17 (0.93)	4.55 (1.19)	−2.250	**<0.001**
Arousal	2.59 (1.81)	4.39 (1.69)	−1.027	**<0.001**
Lubrication	2.86 (2.14)	3.66 (1.65)	−0.417	**0.009**
Orgasm	2.84 (1.92)	3.84 (1.66)	−0.555	**<0.001**
Satisfaction	2.76 (1.42)	4.36 (1.33)	−1.152	**<0.001**
Pain	2.11 (1.95)	2.46 (1.88)	−0.163	0.307
COMPASS-31, mean (s.d.)	54.45 (13.76)	–	–	–
Orthostatic	24.86 (7.58)	–	–	–
Vasomotor	2.66 (1.4)	–	–	–
Secretomotor	6.35 (3.51)	–	–	–
Gastrointestinal (GI)	11.81 (4.19)	–	–	–
Bladder	2.82 (2.24)	–	–	–
Pupillomotor	2.96 (1)	–	–	–
BDI-II, mean (s.d.)	24.61 (11.31)	8.59 (8.24)	1.605	**<0.001**

Significant *P*-values in bold.

### COMPASS-31 scores

In the patient group, the mean COMPASS-31 score was 54.5 (s.d. 13.8). The greatest severity of autonomic symptom burden was recorded in the orthostatic domain [24.9 (s.d. 7.6)], followed by the gastrointestinal [11.8 (s.d. 4.2)] and secretomotor [6.3 (s.d. 3.5)]. The scores overall and across each domain are outlined in Table 1.

### BDI-II scores

Female patients with h-EDS/HSD had significantly higher depression scores than controls. The mean depression score was 24.6 (s.d. 11.4) in the patient group and 8.2 (s.d. 6.8) in controls (Cohen’s *d* = 1.605, *P* ≤ 0.001). The results are outlined in Table 1.

### FSFI scores

**Figure 1.**
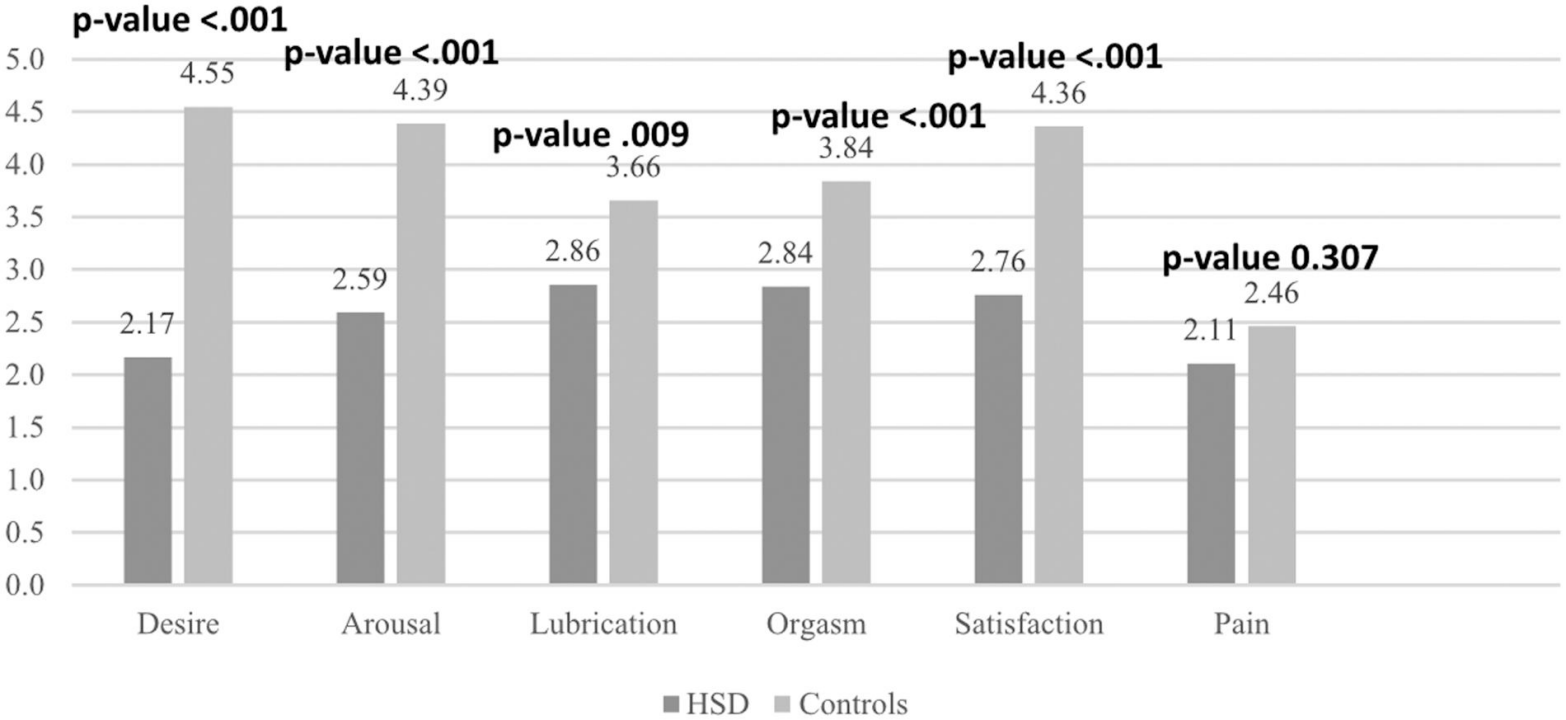
FSFI

The mean sample group FSFI score was 15.34 (s.d. 7.93) in the patient group compared with 23.24 (s.d. 7.21) in controls (Cohen’s *d* = −1.038, *P* ≤ 0.001), as outlined in Table 1. Only 19 (22.6%) women with h-EDS/HSD reported having sexual intercourse with a partner in the past 4 weeks and 2 (2.4%) women reported having sexual intercourse with a partner in the past 3 months (Table 2); in other words, only 25% of patients were sexually active with a partner in the past 6 months. Furthermore, only 38% reported self-stimulation and a majority (52%) did not have sex of any kind in the past 6 months and 18 (21.4%) women reported engaging in no sexual activity of any kind (intercourse or masturbation) in the past 4 weeks. Further data related to activity frequency are outlined in Table 2. The lowest FSFI scores were in the domains of pain [2.11 (s.d. 1.9)] and desire [2.17 (s.d. 0.9)], followed by arousal [2.59 (s.d. 1.8)], satisfaction [2.76 (s.d. 1.4)], orgasm [2.84 (s.d. 1.9)] and lubrication [2.86 (s.d. 2.1)] (Fig. 1). Scores were significantly lower in patients compared with controls across all domains except for pain. Qualitative data revealing patient-perceived reasons for sexual inactivity (Table 2) often related abstinence to chronic pain, fatigue, post-exertional malaise and a lack of libido. The association between sexual dysfunction and h-EDS/HSD persisted when comparing FSFI scores in patients *vs* controls adjusted for age and depression, respectively (Supplementary Tables S1 and S2, available at *Rheumatology Advances in Practice* online).

**Table 2.** Sexual activity and some of the reported reasons for sexual inactivity in women with h-EDS/HSD

Reported having sexual intercourse with partner in different time points, *n* (%)	
Past 4 weeks	19 (22.6)
Past 3 months	2 (2.4)
Past 6 months	0 (0)
Reported self-stimulation in different time points, *n* (%)	
Past 4 weeks	27 (32.1)
Past 3 months	2 (2.4)
Past 6 months	3 (3.6)
Reported both sex with partner and self-stimulation in different time points, *n* (%)	
Past 4 weeks	20 (23.8)
Past 3 months	0 (0)
Past 6 months	0 (0)
No sex of any kind in different time points, *n* (%)	
Past 4 weeks	18 (21.4)
Past 3 months	15 (17.9)
Past 6 months	11 (13.1)
Reported reasons for sexual inactivity	‘Positioning in a comfortable position is difficult for me. My hips pop out of joint, ruining the mood’. ‘I have a very low sex drive. I never crave sex … Sensory issues cause me to be disgusted by soft touching. Sex hurts, not as much since total hysterectomy bit still does. maybe due to tilted uterus?’ ‘Because, to me, it’s not worth the physical pain I will most likely feel during and usually after sexual activity for me to engage in that activity’. ‘I'm too ill to have sex. I haven't been diagnosed with ME as I have long covid, but any exertion triggers PEM [post-exertional malaise]. Plus sex is now painful’. ‘PEM … lack of desire (which was not always the case until like 8 years ago)’. ‘The urge is not there, I have struggled with this my entire life. Haven't had sex in the past 3 years. It is also very painful during intercourse’. ‘Always exhausted, being touched is too painful Lack of interest/drive, lack of energy, low self-esteem, pain during sexual activity’.

### Predictors of sexual dysfunction in h-EDS/HSD

In our patient cohort, multiple linear regression analysis between the FSFI score as the dependent variable and COMPASS-31, age and BDI-II as independent variables was performed. The BDI-II score was correlated to the FSFI score in controls (Table 3), but it was not an independent predictor of sexual function in women with h-EDS/HSD (Tables 4 and 5). The severity of autonomic symptoms (represented by the COMPASS-31 score) did not correlate with FSFI scores in women with h-EDS/HSD (*r* = −0.168, *P* = 0.126). Importantly, sexual dysfunction in women with h-EDS/HSD was associated with age in our cohort (*r* = −0.226, *P = *0.039).

**Table 3.** Pearson correlations of FSFI in h-EDS/HSD patients (*n* = 84).

Group	Age		Depression score		COMPASS-31 Score	
	*r*	*P*-value	*r*	*P*-value	*r*	*P*-value
HSD patients	−0.226	**0.039**	−0.146	0.186	−0.168	0.126
HCs	0.124	0.291	−0.593	**<0.001**	–	–

**Table 4.** Linear regression model for variables predicting sexual dysfunction in h-EDS/HSD patients (*N* = 84)

Variables	Univariable					Multivariable				
	β	SE	95% CI	Std. β	*P*-value	β	SE	95% CI	Std. β	*P*-value
Age	−0.212	0.101	−0.413, −0.011	−0.226	**0.039**	−0.252	0.102	−0.455, −0.049	−0.268	**0.016**
BDI-II	−0.102	0.077	−0.255, 0.050	−0.146	0.186	−0.118	0.08	−0.277, 0.04	−0.169	0.141
COMPASS-31	−0.097	0.063	−0.222, 0.028	−0.168	0.126	−0.074	0.064	−0.2, 0.053	−0.128	0.252

Std.: standardized.

Significant *P*-values in bold.

**Table 5.** Logistic regression model for variables predicting sexual dysfunction in h-EDS/HSD patients (*N* = 84)

Variables	Univariable					Multivariable				
	β	SE	OR	95% CI	*P*-value	β	SE	OR	95% CI	*P*-value
Age	0.113	0.057	1.120	1.001, 1.253	**0.048**	0.136	0.066	1.146	1.007, 1.305	**0.039**
BDI-II	0.001	0.038	1.001	0.930, 1.078	0.981	0.008	0.041	1.008	0.929, 1.093	0.849
COMPASS-31	0.033	0.031	1.034	0.974, 1.098	0.276	0.051	0.039	1.052	0.975, 1.136	0.139

OR: odds ratio.

## Discussion

We found significant sexual dysfunction in women with h-EDS/HSD, which did not correlate with autonomic symptoms or depression. The decreased FSFI scores in the domains of desire, arousal, lubrication, orgasm and sexual satisfaction as compared with HCs, in conjunction with a high prevalence of sexual inactivity, underscore the association and impact of h-EDS/HSD on sexual health.

Importantly, we found that neither depressive nor autonomic symptom burden were predictive of sexual dysfunction in women with h-EDS/HSD. It is likely that given the multifactorial and complex nature of sexual health, multiple physiological and psychosocial factors may play a role in sexual dysfunction that we identified in women with h-EDS/HSD. Compared with patients with POTS from our prior study, patients with h-EDS/HSD reported greater sexual dysfunction overall and across all domains measured in the FSFI (pain with intercourse, satisfaction, orgasm, arousal, desire and lubrication) despite having a similar autonomic symptom burden as reported on COMPASS-31 scores [12]. Additionally, we did not find depression to be a predictive factor of sexual dysfunction in women with h-EDS/HSD, unlike in women with POTS, where depression was identified as a predictive factor for sexual dysfunction. It is possible that the age difference between h-EDS/HSD participants, POTS participants and HCs was a confounding factor, whereas h-EDS/HSD patients were older than both the POTS and HC cohorts. Alternatively, it is possible that pain and not depression was the main driver of sexual dysfunction in h-EDS/HSD patients, but not in patients with POTS. Further studies are needed to determine which factors, alone or in combination, best predict sexual dysfunction and how to mitigate these factors to improve sexual health.

Several factors may have contributed to significant sexual dysfunction observed in h-EDS/HSD patients. The nearly universal presence of chronic pain in our sample (98.8%) likely plays a crucial role in sexual dysfunction. Chronic pain can significantly impair sexual functioning and desire, aligning with the findings of previous studies on chronic pain conditions [13]. Results from this study showed a high prevalence of sexual dysfunction in populations with chronic pain and a positive association between sexual problems and pain severity and psychological concerns. Surprisingly, however, pain during sexual activity was the only FSFI domain that was not significantly different between women with h-EDS/HSD and controls—an unexpected finding considering a nearly 100% prevalence of chronic pain in our cohort and its well-recognized impact on quality of life and sexual function. It is possible that patients did not score high in the pain domain during sexual activity because of the presence of chronic daily pain that affected the responders’ pain perception during sexual activity. Additionally, it is also possible that small fibre neuropathy, which is a common comorbidity in h-EDS/HSD, may have resulted in reduced pain sensation during sexual activity, thereby reducing pain scores to numbers comparable to those in the control group.

Only 25% of patients were sexually active with a partner in the past 6 months and only 38% reported self-stimulation; a majority (52%) did not have sex of any kind in the past 6 months. Fatigue and post-exertional malaise, as reported by our participants, may contribute to reduced sexual activity and satisfaction. Fatigue and malaise can lower motivation as well as physical and emotional capacity for sexual activity, as seen in women with chronic fatigue syndrome whose perception of sexual activity as an overall negative experience correlated with the intensity of fatigue [14]. The possible psychosocial impact of h-EDS/HSD, including pain, anxiety, depression, relationship stress and poor body image, may also contribute to sexual dysfunction. Previous qualitative research has highlighted the emotional and relational strains associated with h-EDS/HSD, which could exacerbate sexual health issues [8].

Future research efforts should concentrate on creating validated, chronic illness–conscious questionnaires to accurately assess sexual health in patients with chronic illness. Moreover, further research is needed to study the hormonal profile in patients with h-EDS/HSD and autonomic disorders to better understand the complex relationship between sex hormones, connective tissue disorders, autonomic dysregulation and sexual dysfunction.

## Limitations

Our study has several limitations. First, the nature of self-reported diagnoses, sexual dysfunction symptoms and recall bias are acknowledged, which may have resulted in imprecise diagnoses and responses. Furthermore, recruitment for participants via social media might have skewed the sample towards more engaged or severe cases, which could affect the generalizability of our findings to a broader population. Second, the patients were somewhat older than controls, which may affect direct comparisons and conclusions about independent variables. Third, in this study, we did not assess medications and its possible sexual dysfunction side effects: this assessment is best conducted in the context of a large, multicentre prospective study, especially since h-EDS/HSD are rare disorders and therefore recruitment of a large cohort might be difficult. Fourth, hereditary connective tissue disorders likely encompass a broad spectrum of manifestations and phenotypes, which means that some patients who reported being diagnosed with h-EDS may have had HSD and vice versa. Finally, despite being a widely used and validated tool to determine sexual dysfunction in healthy women, the validity of the FSFI in patients with chronic disorders is presently unknown. Taken together, these limitations restrict the ability to draw robust conclusions; estimates should not be interpreted causally and should be considered as exploratory findings that require future confirmation. Despite these limitations, however, our study provides the first and only evidence available to date of sexual dysfunction in women with h-EDS/HSD in a sizable cohort of patients.

## Conclusion

Compared with healthy women, women with h-EDS/HSD experience significant sexual dysfunction and sexual inactivity. Autonomic dysfunction or depressive symptoms do not appear to be predictive of sexual dysfunction in women with h-EDS/HSD. Further research examining sexual function, hormone levels and other factors relevant to sexual health is needed to determine the complex and multifactorial aetiology of sexual dysfunction in people with h-EDS/HSD. Given its significant implications in reproduction, relationships, family planning and overall health and well-being, sexual dysfunction represents a significant unmet need that calls for development of targeted multidisciplinary diagnostic and therapeutic approaches in the care of patients with h-EDS/HSD.

## Supplementary Material

rkaf023_Supplementary_Data

## Data Availability

The data underlying this article will be shared upon reasonable request to the corresponding author.
