# Supplementary material for: Sexual dysfunction in women with hypermobile Ehlers–Danlos syndrome and hypermobility spectrum disorders: an online community-based study
Source: Rheumatol Adv Pract. 2025 Feb 27;9(2):rkaf023. doi: 10.1093/rap/rkaf023 (PMC11964486; doi:10.1093/rap/rkaf023)
Supplement: rkaf023_Supplementary_Data [file rkaf023_supplementary_data.zip › 24-239 Supplementary Material.docx]

**SUPPLEMENTARY MATERIAL**

**Supplementary Table S1**. Comparing sexual function, adjusted for age, between h-EDS/HSD patients and healthy controls using linear regression

|  | h-EDS/HSD Patients (n = 84) | Healthy Controls (n = 75) | F | **P-value** |
| --- | --- | --- | --- | --- |
| **FSFI; mean (SD)** | 15.34 (7.93) | 23.24 (7.21) | 27.2 | **<0.001** |
| Desire | 2.17 (0.93) | 4.55 (1.19) | 137.85 | **<0.001** |
| Arousal | 2.59 (1.81) | 4.39 (1.69) | 23.8 | **<0.001** |
| Lubrication | 2.86 (2.14) | 3.66 (1.65) | 3.68 | **0.057** |
| Orgasm | 2.84 (1.92) | 3.84 (1.66) | 7.52 | **0.007** |
| Satisfaction | 2.76 (1.42) | 4.36 (1.33) | 32.39 | **<0.001** |
| Pain | 2.11 (1.95) | 2.46 (1.88) | 1.45 | **0.231** |

**Supplementary Table S2**. Comparing sexual function, adjusted for depression, between h-EDS/HSD patients and healthy controls using linear regression

|  | h-EDS/HSD Patients (n = 84) | Healthy Controls (n = 75) | F | **P-value** |
| --- | --- | --- | --- | --- |
| **FSFI; mean (SD)** | 15.34 (7.93) | 23.24 (7.21) | 7.72 | **0.006** |
| Desire | 2.17 (0.93) | 4.55 (1.19) | 80.95 | **<0.001** |
| Arousal | 2.59 (1.81) | 4.39 (1.69) | 9.03 | **0.003** |
| Lubrication | 2.86 (2.14) | 3.66 (1.65) | 0.185 | **0.668** |
| Orgasm | 2.84 (1.92) | 3.84 (1.66) | 0.54 | **0.463** |
| Satisfaction | 2.76 (1.42) | 4.36 (1.33) | 10.65 | **0.001** |
| Pain | 2.11 (1.95) | 2.46 (1.88) | 0.046 | **0.83** |
